# Editorial: Significance of Cellular Lipids for Viral Replication and Pathogenesis

**DOI:** 10.3389/fphys.2022.906205

**Published:** 2022-04-28

**Authors:** Ulrich Desselberger, Carolina Henritta Pohl, Hester Gertruida O’Neill

**Affiliations:** ^1^ Department of Medicine, University of Cambridge, Cambridge, United Kingdom; ^2^ Department of Microbiology and Biochemistry, University of the Free State, Bloemfontein, South Africa

**Keywords:** lipid droplets, sphingolipids, cholesterol, viral replication, viral entry, broadspectrum antiviral therapy

Cellular lipids and their metabolism have increasingly been recognized as essential for various steps of viral replication cycles, including viral entry, genome transcription and replication, particle assembly and egress. These cellular lipids include various structural molecules, such a gangliosides, phospholipids, sterols and sphingolipids, and are localized in cellular membranes and lipid droplets (LDs). Alterations of lipid homeostasis can promote or block viral replication and are often accompanied by the production of pro-inflammatory cytokines. Therefore, cellular lipids and their metabolites are considered to be attractive targets for the development of new broad-spectrum antiviral therapies.

The biosynthesis of intracellular fatty acids and sterols as well as their metabolic pathways are well researched (Walther and Farese, 2012; Crawford and Desselberger, 2016). Lipid droplets (LDs) are major storage organelles of neutral lipids, such as triacylglycerols and cholesterol esters, and act as vehicles for intracellular transport (Walther et al., 2017). These organelles, and other lipids, are also important for the replication of various RNA viruses such as hepatitis C viruses, Dengue viruses (Chatel-Chaix and Bartenschlager, 2014; Bang et al., 2019), species A rotaviruses (Cheung et al., 2010), picornaviruses (Belov and van Kuppeveld, 2019; Laufman et al., 2019), noroviruses (Doerflinger et al., 2017), SARS-CoV-2 (Dias et al., 2020; Nardacci et al., 2021; Theken et al., 2021) and some DNA viruses such as Marek’s disease virus (Boodhoo et al., 2019). However, the specific mechanisms by which these viruses interact with the cellular lipidome remain incompletely understood.

The roles of cellular lipids in viral replication and pathogenesis and their potential to be developed as drug targets were highlighted in seven articles in the Research Topic by *Frontiers in Physiology*.

Avota et al. assessed the various roles played by sphingolipids during the different steps of viral replication (virus attachment and entry, viral replication, morphogenesis and budding), updating earlier reviews (Schneider-Schaulies and Schneider-Schaulies, 2015; Schneider-Schaulies et al., 2021). They also discuss the possibility to repurpose inhibitors of sphingolipid metabolism already in clinical use for treatment of viral infections.

Similarly, Farfan-Morales et al. discuss the use of FDA-approved lipid-lowering drugs as host cell-targeted antivirals against flavivirus disease caused by Dengue and Zika viruses. In this context the contribution by Vial et al. is of interest. In mosquitoes, which act as vectors for various human flavivirus infections, the phospholipidome is upregulated during virus replication. This finding may lead to the development of novel, host-specific antivirals with the aim to block transmission to the human host.

Other researchers highlighted the potential of various lipid metabolic pathways as antiviral targets. Cellular cholesterol, as a component of cellular lipid rafts has recently gained much attention as being required for cellular entry of many enveloped and non-enveloped viruses (Ripa et al., 2021; Sanders et al., 2021). In the review by Glitscher and Hildt, the fact that viruses redistribute cellular cholesterol during infection was emphasised. Furthermore, it was proposed that strategies to manage the distribution of cellular cholesterol during viral infection, rather than a global reduction of cholesterol metabolism, be investigated for the development of broad-spectrum antivirals. Measles virus replication in primary human lymphocytes was found to be significantly reduced by inhibitors of ceramidase and sphingosine kinases 1 and 2 (Chithelen et al.), which correlated with the blockage of the phosphorylation of cellular translation factors.

Lipid droplets are important for rotavirus replication and take part in the formation of viral factories, termed viroplasms (Cheung et al., 2010). Crawford et al. reported that a recombinant rotavirus A strain containing a mutant NSP2 protein exhibits delayed viroplasm formation. They observed an early interaction of NSP2 with phosphorylated perilipin 1 located on LDs, thus dissecting part of the molecular mechanism of viroplasm-LD interaction during rotavirus replication. Following rotavirus infection, Sander et al. observed an increase in the production of the arachidonic acid metabolite, the eicosanoid prostaglandin E_2_. This metabolite has been implicated in various steps of viral replication, including viral binding to cellular receptors, viral gene expression, increase in pro-inflammatory cytokines and the production and release of nascent virions (Sander et al., 2017). In the current study, co-localization between prostaglandin E_2_ and viroplasms was shown. Inhibitors of prostaglandin E_2_ synthesis, such as indomethacin, reduced the yield of infectious viral progeny by approximately 1 log step. This finding points to a role of prostaglandin E_2_ in enhancement of rotavirus attachment and internalisation.

Viral factories have recently been recognized as liquid-liquid phase separation (LLPS)-driven protein-RNA condensates (Etibor et al., 2021; Geiger et al., 2021; Papa et al., 2021) which change their material properties over time and can interact with LDs at later stages of infection. Details of the interaction of the biomolecular condensates with cellular compounds (LDs, tubulin, and others) remain to be elucidated.

Up to now, the interaction of the cellular lipidome with replication steps of mammalian viruses has been explored mainly *in vitro*. Translational research of these relationships in intestinal organoids (Saxena et al., 2015; Ettayebi et al., 2016) and suitable animal models is very much at the beginning but is promising for the development of broad-spectrum antiviral therapy. However, this pathway may not always be straightforward. In the search of licensed drugs for repurposing to treat COVID-19 disease, it was recently found that most of the drugs with antiviral activity were cationic amphiphilic compounds associated with the development of phospholipidosis in cells and organs, thus confounding this avenue of drug discovery (Tummino et al., 2021). While interference with lipid metabolism may hold the potential for the development of broad-spectrum antiviral therapies, this is a complex approach which will require further in-depth basic research.

## References

[B1] Bang B.-R. Li M. Tsai K.-N. Aoyagi H. Lee S.-A. Machida K. (2019). Regulation of Hepatitis C Virus Infection by Cellular Retinoic Acid Binding Proteins through the Modulation of Lipid Droplet Abundance. J. Virol. 93 (8), e02302–18. 10.1128/JVI.02302-18 30728260 PMC6450116

[B2] Belov G. A. van Kuppeveld F. J. M. (2019). Lipid Droplets Grease Enterovirus Replication. Cell Host & Microbe 26 (2), 149–151. 10.1016/j.chom.2019.07.017 31415743 PMC9004532

[B3] Boodhoo N. Kamble N. Kaufer B. B. Behboudi S. (2019). Replication of Marek's Disease Virus Is Dependent on Synthesis of De Novo Fatty Acid and Prostaglandin E 2. J. Virol. 93 (13), e00352–19. 10.1128/JVI.00352-19 30971474 PMC6580946

[B4] Chatel-Chaix L. Bartenschlager R. (2014). Dengue Virus- and Hepatitis C Virus-Induced Replication and Assembly Compartments: the Enemy Inside-Caught in the Web. J. Virol. 88 (11), 5907–5911. 10.1128/JVI.03404-13 24623440 PMC4093888

[B5] Cheung W. Gill M. Esposito A. Kaminski C. F. Courousse N. Chwetzoff S. (2010). Rotaviruses Associate with Cellular Lipid Droplet Components to Replicate in Viroplasms, and Compounds Disrupting or Blocking Lipid Droplets Inhibit Viroplasm Formation and Viral Replication. J. Virol. 84 (13), 6782–6798. 10.1128/JVI.01757-09 20335253 PMC2903253

[B6] Crawford S. E. Desselberger U. (2016). Lipid Droplets Form Complexes with Viroplasms and Are Crucial for Rotavirus Replication. Curr. Opin. Virol. 19, 11–15. 10.1016/j.coviro.2016.05.008 27341619 PMC5125616

[B7] Dias S. S. G. Soares V. C. Ferreira A. C. Sacramento C. Q. Fintelman-Rodrigues N. Temerozo J. R. (2020). Lipid Droplets Fuel SARS-CoV-2 Replication and Production of Inflammatory Mediators. Plos Pathog. 16 (12), e1009127. 10.1371/journal.ppat.1009127 33326472 PMC7773323

[B8] Doerflinger S. Y. Cortese M. Romero-Brey I. Menne Z. Tubiana T. Schenk C. (2017). Membrane Alterations Induced by Nonstructural Proteins of Human Norovirus. Plos Pathog. 13 (10), e1006705. 10.1371/journal.ppat.1006705 29077760 PMC5678787

[B9] Etibor T. Yamauchi Y. Amorim M. (2021). Liquid Biomolecular Condensates and Viral Lifecycles: Review and Perspectives. Viruses 13 (3), 366. 10.3390/v13030366 33669141 PMC7996568

[B10] Ettayebi K. Crawford S. E. Murakami K. Broughman J. R. Karandikar U. Tenge V. R. (2016). Replication of Human Noroviruses in Stem Cell-Derived Human Enteroids. Science 353 (6306), 1387–1393. 10.1126/science.aaf5211 27562956 PMC5305121

[B11] Geiger F. Acker J. Papa G. Wang X. Arter W. E. Saar K. L. (2021). Liquid-liquid Phase Separation Underpins the Formation of Replication Factories in Rotaviruses. EMBO J. 40 (21), e107711. 10.15252/embj.2021107711 34524703 PMC8561643

[B12] Laufman O. Perrino J. Andino R. (2019). Viral Generated Inter-organelle Contacts Redirect Lipid Flux for Genome Replication. Cell 178 (2), 275–289. e16. 10.1016/j.cell.2019.05.030 31204099 PMC7077330

[B13] Nardacci R. Colavita F. Castilletti C. Lapa D. Matusali G. Meschi S. (2021). Evidences for Lipid Involvement in SARS-CoV-2 Cytopathogenesis. Cell Death Dis 12 (3), 263. 10.1038/s41419-021-03527-9 33712574 PMC7952828

[B14] Papa G. Borodavka A. Desselberger U. (2021). Viroplasms: Assembly and Functions of Rotavirus Replication Factories. Viruses 13 (7), 1349. 10.3390/v13071349 34372555 PMC8310052

[B15] Ripa I. Andreu S. López-Guerrero J. A. Bello-Morales R. (2021). Membrane Rafts: Portals for Viral Entry. Front. Microbiol. 12, 631274. 10.3389/fmicb.2021.631274 33613502 PMC7890030

[B24] Sander W. J. O’Neill H. G. Pohl C. H., (2017). Prostaglandin E2 as a modulator of viral infections. Front. Physiol. 8:89. 10.3389/fphys.2017.00089 PMC5306375 28261111

[B16] Sanders D. W. Jumper C. C. Ackerman P. J. Bracha D. Donlic A. Kim H. (2021). SARS-CoV-2 Requires Cholesterol for Viral Entry and Pathological Syncytia Formation. Elife 10, e65962. 10.7554/eLife.65962 33890572 PMC8104966

[B17] Saxena K. Blutt S. E. Ettayebi K. Zeng X.-L. Broughman J. R. Crawford S. E. (2016). Human Intestinal Enteroids: a New Model to Study Human Rotavirus Infection, Host Restriction, and Pathophysiology. J. Virol. 90 (1), 43–56. 10.1128/JVI.01930-15 26446608 PMC4702582

[B18] Schneider-Schaulies J. Schneider-Schaulies S. (2015). Sphingolipids in Viral Infection. Biol. Chem. 396 (6-7), 585–595. 10.1515/hsz-2014-0273 25525752

[B19] Schneider-Schaulies S. Schumacher F. Wigger D. Schöl M. Waghmare T. Schlegel J. (2021). Sphingolipids: Effectors and Achilles Heals in Viral Infections? Cells 10 (9), 2175. 10.3390/cells10092175 34571822 PMC8466362

[B20] Theken K. N. Tang S. Y. Sengupta S. FitzGerald G. A. (2021). The Roles of Lipids in SARS-CoV-2 Viral Replication and the Host Immune Response. J. Lipid Res. 62, 100129. 10.1016/j.jlr.2021.100129 34599996 PMC8480132

[B21] Tummino T. A. Rezelj V. V. Fischer B. Fischer A. O’Meara M. J. Monel B. (2021). Drug-induced Phospholipidosis Confounds Drug Repurposing for SARS-CoV-2. Science 373 (6554), 541–547. 10.1126/science.abi4708 34326236 PMC8501941

[B22] Walther T. C. Chung J. Farese R. V. Jr (2017). Lipid Droplet Biogenesis. Annu. Rev. Cel Dev. Biol. 33, 491–510. 10.1146/annurev-cellbio-100616-060608 PMC6986389 28793795

[B23] Walther T. C. Farese R. V. Jr (2012). Lipid Droplets and Cellular Lipid Metabolism. Annu. Rev. Biochem. 81, 687–714. 10.1146/annurev-biochem-061009-102430 22524315 PMC3767414

